# Advert saliency distracts children's visual attention during task-oriented internet use

**DOI:** 10.3389/fpsyg.2014.00051

**Published:** 2014-02-12

**Authors:** Nils Holmberg, Helena Sandberg, Kenneth Holmqvist

**Affiliations:** ^1^Department of Communication and Media, Lund UniversityLund, Sweden; ^2^Lund University Humanities Lab, Lund UniversityLund, Sweden

**Keywords:** online advertising, children, internet use, distraction, visual saliency, visual attention

## Abstract

The general research question of the present study was to assess the impact of visually salient online adverts on children's task-oriented internet use. In order to answer this question, an experimental study was constructed in which 9- and 12-year-old Swedish children were asked to solve a number of tasks while interacting with a mockup website. In each trial, web adverts in several saliency conditions were presented. By both measuring children's task accuracy, as well as the visual processing involved in solving these tasks, this study allows us to infer how two types of visual saliency affect children's attentional behavior, and whether such behavioral effects also impacts their task performance. Analyses show that low-level visual features and task relevance in online adverts have different effects on performance measures and process measures respectively. Whereas task performance is stable with regard to several advert saliency conditions, a marked effect is seen on children's gaze behavior. On the other hand, task performance is shown to be more sensitive to individual differences such as age, gender and level of gaze control. The results provide evidence about cognitive and behavioral distraction effects in children's task-oriented internet use caused by visual saliency in online adverts. The experiment suggests that children to some extent are able to compensate for behavioral effects caused by distracting visual stimuli when solving prospective memory tasks. Suggestions are given for further research into the interdiciplinary area between media research and cognitive science.

## 1. Introduction

Children's internet use is known to vary a lot between countries (Holloway et al., 2013). In Sweden, children attending primary school and middle school come into contact with the internet in a wide variety of everyday situations, ranging from information search in connection to school projects, to instant messaging on mobile phones during leisure activities. Current media research indicates that children spend an increasing amount of time connected to the internet, and that “tweens” aged between 9 and 12 spend about 1–2 h online a day on average. Notably, there is a steep increase in online activities between these two age groups, and time spent online per day is more than doubled over this age interval (Nordicom, 2013). Typical online activities among 9-year-old children are playing games and watching video clips. These activities are also found among 12-year-olds, but in addition there is a pronounced increase in time spent on social networking websites (Findahl, 2012).

Online advertising seems to quickly become a natural and persistent part of children's overall online experience. Interview studies of 9-year-old children show that, while online advertising is generally perceived as disturbing and confusing, these adverts are also largely tolerated and even sometimes consumed as entertainment (Martinez et al., 2013). However, at the same time these children often show a naive conception about the commercial and persuasive intent of online advertising, as well as a limited understanding of how online advertising use visual cues to capture attention (Buijzen et al., 2010). Whereas older children naturally start to develop the necessary attentional mechanisms needed to shield off impinging visual stimuli from online adverts, these neural structures are still developing in younger children, causing them to react to salient adverts on an involuntary level (Kramer et al., 2005). Involuntary exposure to online advertising might be a problem during free entertainment-based web surfing (depending on whether adverts are age-appropriate, distinct, recognizable etc.), but such “forced exposure” could become much more problematic in the case of task-oriented internet use. In the latter scenario, online adverts could introduce a disruptive element in situations where children are trying to pursue goal-directed activities online. Should this be the case, it becomes urgent to safeguard young children's rights to equal opportunities online (Holloway et al., 2013).

In order to investigate younger children's cognitive sensitivity to online advertising, we believe it is crucial to take into account both individual factors such as age, gender and cognitive development, as well as the visual properties of online adverts. In this study we used the anti-saccade task to determine children's individual level of oculomotor control. This paradigm directly measures participants' voluntary control of their eye movements, and has been shown to correlate with cognitive functions such as executive control, working memory capacity and visual distractibility (Munoz and Everling, 2004, Kramer et al., 2005, Zanelli et al., 2005, Hutton and Ettinger, 2006). Several studies have also shown that this capacity to inhibit stimulus-driven, reflexive eye movements undergoes significant development throughout childhood, which is linked to increased development of the frontal lobe (Klein and Foerster, 2001, Eenshuistra et al., 2004). To our knowledge, there have been no attempts to measure childrens' visual distractibility in relation to their advertising exposure. In the present study, the motivation for measuring oculomotor control in two age-groups was that we intended to differentiate the effects of prefrontal control from other age-related effects, and that we wanted to find out if better gaze control is related to less advert distraction. The motivation for selecting age groups at 9 and 12 years was that previous research has shown that these ages respresent clear developmental and cognitive stages in children's understanding of persuasive advertising content (Buijzen et al., 2010).

In a recent study, we analyzed low-level saliency features in internet adverts with regard to children's visual attention (Holmberg et al., 2013). In this study, a group of 9-year-old children were allowed to surf freely on their favorite websites, while eye movement data were collected along with real-time screen recordings of the web page stimuli. These screen recordings were used to quantify low-level saliency aspects in all adverts that the children encountered. Key findings of this exploratory study were: (1) that low-level saliency features such as motion (pixel change), luminance and edge density in online adverts had a positive correlation with children's visual attention, and (2) that children with low individual level of gaze control had an increased sensitivity to these saliency features. Other studies have focused on stimulus onset as a key component in low-level visual saliency. This research has shown that abrupt onset of visual stimuli has a powerful effect on attentional capture (Ludwig et al., 2008), and that such low-level factors can impair task performance by distracting attention and increasing cognitive load (Lavie, 2005). There is also some evidence of “high-level saliency features” and their effects on attention (Findlay and Walker, 1999). High-level saliency refer to visual features that become relevant depending on the subject's particular cognitive task (Malcolm and Henderson, 2010), and in order to avoid confusion we will refer to this kind of saliency as “task relevance.” Currently, there is a fairly strong consensus that, while low-level visual features such as abrupt onset can account for a some portion of people's eye movement behavior, task relevance is more powerful in explaining visual attention allocation (Foulsham and Underwood, 2008, Tatler et al., 2011).

The general research question of the present study was to assess the impact of internet adverts on children's internet use. Since internet use is a fairly broad concept including several types of interaction, we decided to focus further on one particular type of use case in which children interact with web pages in order to solve a predefined task involving memory and judgement. This type of web interaction should have a high intrinsic value to children, and should consequently be facilitated rather than hindered by the commercial online environments that children encounter. We reasoned that distraction caused by internet adverts would affect both the gaze behavior involved in the process of solving the tasks, as well as the task performance. In order to create experimental manipulations of the internet adverts, we utilized two aspects of visual saliency that are well-known in vision research: low-level visual features (Itti and Koch, 2000; Peters et al., 2005) and content relevance (Henderson, 2003). Low-level visual features were manipulated by varying the advert onset speed, and this feature was expected to distract the visual processing by attracting visual attention to the adverts (measured as saccades to ads). Advert relevance was manipulated by varying the level of task relevance in the advert content, and was expected to cause visual distraction by retaining attention on the adverts (measured as dwell time on ads).

Users' visual interaction with web pages and other interfaces will differ widely depending on the particular task that the interaction process is intended to solve (e.g., Yarbus, 1967; Cowen et al., 2002). The more viewers are allowed to decide their own subjective goals during visual interaction, the more these viewing patterns will vary between individuals (so-called free viewing conditions, e.g., Jansen et al., 2009). By contrast, if viewers are presented with a distinct and uniform task, viewing patterns will generally show much more similarities. In behavioral and psychological research the latter case usually means that it becomes easier to detect weak behavioral signals among random noise. It has been shown that in the absence of a task, viewing patterns become more influenced by low-level saliency features, and conversely, if a task is present, viewing patterns become more concentrated to task-relevant visual features (Hooge et al., 2005). An additional benefit from using a task-oriented experimental paradigm is that the visual interaction process can be evaluated in terms of performance, where some interaction strategies can be linked to better outcomes. In a free viewing task such evaluation of the interaction process is much more difficult. Finally, a task-oriented paradigm is also sensitive to the subjects individual level of expertise, and thus it is possible to isolate and estimate the positive effect of task expertise on solving a particular task (Jarodzka et al., 2010).

By constructing an experimental website that repeatedly presented children with a series of similar tasks, the present study has sought to benefit from all the positive aspects of task-oriented study designs previously mentioned. Thus we expected to find a high overall attentional focus on task relevant elements on the web pages, as well as a difference between younger and older children (caused by a higher level of internet expertise in the latter group). But more importantly, by using a task-oriented paradigm we expect to find differences in task performance depending on the advert saliency manipulations presented in each trial. Task performance is both measured through *performance measures* involving the accuracy and duration of each task response, but also as several *distraction measures* describing the children's visual interaction with the web pages. Thus, our experiment allowed us to test the effects of advert saliency conditions on performance measures and on distraction measures respectively, and it also allowed us to explore possible links between these two kinds of measures. This is crucial since advert saliency might affect these measures differently, and in that case it is important to be able to capture these differential effects to get a correct understanding of the effects of advertising saliency on children's task-oriented internet use.

Two types of performance measures were assumed to be sensitive to advert saliency manipulations: *trial accuracy* and *trial duration*. We reasoned that the advert saliency conditions would distract the children and cause a lower ability to solve the tasks correctly (trial accuracy) and efficiently (trial duration). For the sake of simplicity, high trial accuracy and low trial duration are grouped together as high task performance in the hypotheses. We hypothesized directional effects on task performance caused by the following experimental factors:

- H1a: Higher age and gaze control in children will be related to higher task performance.
- H1b: Higher onset speed in adverts will cause lower task performance.
- H1c: Higher task relevance in adverts will cause lower task performance.

Two types of distraction measures were constructed in order to capture effects of the advert saliency manipulations: *saccades to ads* and *dwell time on ads*. Saccades to ads measure the number of times visual attention has been shifted toward experimental adverts instead of objects relevant for solving the tasks. We reasoned that this measure would capture one important aspect of distraction: (1) the attention attracting power of ads. Dwell time on ads measure the actual amount of time spent on experimental adverts instead of other objects that are critical for solving the tasks, and we reasoned that this measure would capture a second crucial aspect of distraction: (2) the attention retaining power of ads. For simplicity, these two aspects of distraction are grouped together in the hypotheses. We hypothesized directional effects on distraction measures caused by the following experimental factors:

- H2a: Higher age and gaze control in children will be related to less task distraction.
- H2b: Higher onset speed in adverts will cause more task distraction.
- H2c: Higher task relevance in adverts will cause more task distraction.

As can be deduced from the hypotheses listed above, the current study contains both correlational hypotheses (H1a and H2a) as well as more causal hypotheses associated with experimental manipulations (H1b, H1c, H2b, and H2c). This structure will also be reflected when presenting and discussing the results of the study.

## 2. Materials and methods

### 2.1. Participants and apparatus

The participants were selected from two age groups, 9-year-olds (*n* = 19) and 12-year-olds (*n* = 26), and were recruited from an elementary school in the south of Sweden. The distribution was fairly equal between girls (*n* = 23) and boys (*n* = 22). Only children that were given parental consent participated in the study (*n* = 45). The data recording equipment consisted of an SMI RED 250 eye-tracking camera and a laptop computer (Intel Core i7 2.67 GHz CPU, 2.98 GB RAM). The laptop was used both for stimulus presentation and eye movement recordings, and was connected to the Internet through a wireless 4 G router. Visual stimuli were presented on a 1680 × 1050 LCD monitor. The interactive web tasks were presented using the standard Internet Explorer 8 web browser. Eye-tracking data were recorded at 250 Hz using the SMI iViewX 2.7 software during all experimental modules.

### 2.2. Experimental design and materials

A pre-test was administered to all children in the form of an anti-saccade test. After 4 practice trials, a series of 32 anti-saccade trials were presented to each participant. In each trial, a central fixation cross was replaced by a peripheral target, and participants were instructed to look in the opposite direction relative to the target location. The stimulus parameters of the anti-saccade test were chosen in accordance with recently suggested standards (Antoniades et al., 2013). Thus, the test was designed with no temporal gap between central fixation offset and target onset. The central fixation foreperiod was set to a duration of 1500–2000 ms, and the target duration was set to 1000 ms. After target offset, a blank screen was presented for 500 ms. Targets were presented in four randomized locations (top, bottom, left, and right) with an amplitude of ca 10° from the central fixation cross. To reduce fatigue in the children, the stimuli were constructed with a dark background.

**Figure 1:**
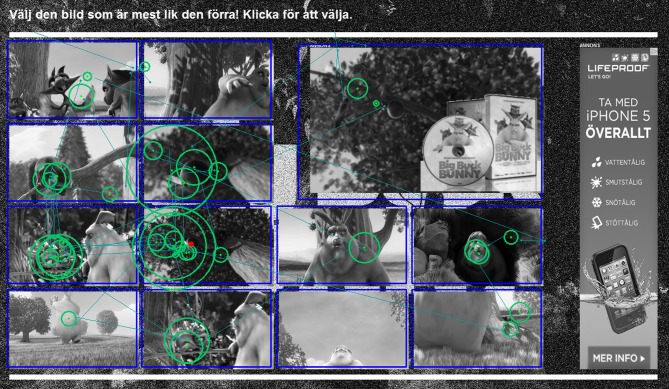
**Layout of the image selection web page used to solve the online tasks.** The image array contains 3 target images and 9 distractor images in randomized positions. An experimental advert in the high task relevance condition is presented in the top-right corner. The banner advert to the far right was added to make the web pages more realistic, and was kept constant in all trials. The participant's eye movements during the trial are superimposed on the web page image.

Children in both age groups performed the exact same experiment, which consisted of 36 trials. Each trial consisted of a web-based visual search task, in which the participant was instructed to memorize a single image presented on an initial web page, and then proceed to a second web page to select the the most similar image in an array of 12 images. On the second page, 3 similar but unique target images were presented, along with 9 unique distractor images with lower similarity (Figure 1). The experimental images were created by splitting an animated open source movie (Big Buck Bunny, © 2008, Blender Foundation) into separate frames, and image similarities were determined using the OpenCV histogram comparison algorithm (Bradski, 2000). Target images were selected from a high correlation coefficient interval (0.95 ≥ *r* ≥ 0.65), while distractor images were selected using a lower threshold (*r* ≤ 0.10). An important implication of this image similarity approach was that target images were never identical to the initial image, which added a cognitive component due to the fact that finding an optimal solution encouraged the participants to perform an image similarity judgement. The initial image memorization phase was self-paced, while the second image selection phase introduced a 7000 ms delay before the web page allowed the participant to select an image and thus move to the next web task.

**Figure 2:**
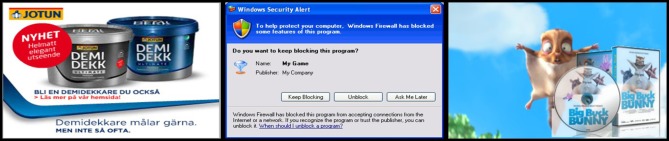
**In the task relevance conditions, the pictorial content of the adverts was manipulated to different degrees of task relevance.** On the left hand side, an irrelevant advert in the control condition is shown. In the middle and on the right hand side, advert content in the low task relevance and high task relevance conditions is shown respectively. In the latter condition, the advert were always based on the target image of the task. (Reprinted with permission.)

On the second image selection web page, an online advert was presented according to 9 saliency conditions. The low-level saliency conditions were operationalized as two levels of advert onset speed, which were implemented as animated GIF images. Each GIF animation consisted of a number of transitional frames between the advert image and a blank white image, and was presented at a frame rate of 10 fps. Smooth advert onset was created using 50 transitional frames and a 1000 ms pause, while abrupt onset speed was created using 2 transitional frames and a 3000 ms pause. The GIF animations were then looped in order to present the low-level saliency conditions continuously on the web pages during each trial. The onset speed manipulation gave the visual impression that the adverts disappeared and then reappeared softly or abruptly on the web pages (Supplementary Material). The onset speed factor also included a control condition, consisting of the static advert images. These low-level saliency conditions were then combined with three types of task relevance including a control condition, producing a total of 3 × 3 advert saliency conditions. The task relevance conditions were operationalized as two levels of task relevant pictorial content in adverts. Adverts in the low task relevance condition depicted system dialog windows and website login windows, while adverts in the high task relevance condition depicted mockup adverts that closely resembled the target pictures in tasks (Figure 2). The task relevance factor also included a control condition depicting irrelevant inanimate objects.

The advert conditions were presented in randomized order during the web-based tasks. Each advert saliency condition was repeated four times, and advert positions were randomized between the four corners of the web page (top-left, top-right, bottom-left, and bottom-right). These positions were assumed to emulate typical advertising positions on real web pages. All adverts except those in the high task relevance condition were based on naturally occurring adverts found on websites frequently used by children in the current sample.

### 2.3. Procedure

Each child was first greeted and presented with a verbal outline of the web-based tasks to be performed. Careful consideration was taken to ensure that the children were kept naive about the focus on online adverts and the exact nature of our data recordings. Prior to all eye-tracking recordings the participants were calibrated using a 5-point calibration method available in the SMI iViewX software. Calibrations were done at an eye-monitor distance of ca 700 mm, and were repeated until the horizontal and vertical deviation was below 1° of visual angle. After the first calibration, each participant underwent a 9 plate Ishihara color vision test presented on screen (Hardy et al., 1945). Results of this test indicated that all participants had full color vision. After another calibration, an anti-saccade test was performed containing 4 initial test trials and 32 actual trials. A third calibration was then undertaken before a web browser loaded and presented the instructions for the web-based tasks. First, the participants were instructed on how to solve the tasks through a detailed verbal walk-through of two test trials. The participants were instructed to memorize an initial image for each task and then try to find and click on the most similar image on a second web page. No information was given about the number of target and distractor images. Instructions were given to complete each task as accurately as possible, rather than as quickly as possible. The participants were not given any information about the advert content accompanying each task, and thus they were not instructed to avoid any adverts. All participants received a movie ticket as reward for active participation in the study. When the data collection phase was finished, meetings were arranged with the children in order to inform them about the true purpose and methods of the experiment.

### 2.4. Data analysis

The overall quality of the eye-tracking data was calculated as the average deviation between the calibrated point of regard (POR) and 4 validation points. The average horizontal and vertical deviation was 0.75 and 0.92° respectively. The amount of missing samples (including blinks) in the anti-saccade data was 12.6%. These quality measures were only calculated for the anti-saccade dataset, but it should generalize to the dataset for internet use as well, since the exact same calibration procedure was applied in both cases.

Eye movement data from the anti-saccade test were analyzed by using the Engbert and Kliegel algorithm in order to detect the first saccade in each trial (Engbert and Kliegl, 2003). A minimum saccade duration of 32 ms was provided as a parameter for the detection algorithm. The first saccades were then analyzed for latency, peak velocity and direction relative to target position using a second algorithm (Ahlström et al., 2013). Saccade latency was calculated using a minimum latency parameter of 0.08 ms, peak velocity was calculated using a maximum saccade velocity parameter of 1000°/s. Anti-saccades were categorized binomially as correct if they were terminated within a 45° angle in the opposite direction of the target location. Only the total proportion of correct anti-saccades for each participant was used for further analysis, as this construct was considered to be the most valid measure of gaze control.

Behavioral data from the children's task-oriented internet use were analyzed in two major steps. In the first step, the two performance measures were analyzed. Trial accuracy was determined by analyzing mouse click responses recorded by SMI Experiment Center 3.2 and encoding these responses as a binomial variable depending on whether the tasks had been solved correctly by clicking on one of the target images. The second product measure, trial duration, was also analyzed in this step by recording the time difference in milliseconds between trial onset (when the task web pages were loaded) and the participants' response (when the mouse click was used to solve the task). Since the SMI software logged the timing of these events, we could control for variable network latencies in the web-based stimulus presentation.

In the second step, the eye movement data from each trial were extracted and eye movement events such as fixations, saccades and blinks were detected using the SMI BeGaze 3.2 software. These event detected eye movement data were then used to calculate the two distraction measures in relation to the area of interest (or AOI) corresponding to the adverts. Dwell time on ads was calculated by adding all fixation durations on the experimental ads for each trial. This AOI-based measure is better known as *total dwell time* in the eye-tracking literature (Holmqvist et al., 2011). The function of this measure is often to provide a close approximation of the total amount of visual attention devoted to a specific region in the visual field. Saccades to ads were calculated by counting the number of saccades that originated outside the pixel coordinates of the experimental advert AOIs, and terminated inside this same region (a variation of the more common *number of saccades* and *number of transitions* measures) (Holmqvist et al., 2011). The denomination of the distraction measures was chosen in order to clearly contrast the functional difference between fixations and saccades. Thus, saccades to ads were assumed to measure the ads' attention attracting power, while dwell time on ads was assumed to measure their attention retaining power (Born and Kerzel, 2008).

## 3. Results

### 3.1. Children's gaze control

The children's individual level of gaze control was measured with an anti-saccade test in the beginning of the experiment. The proportion of trials containing valid eye movement data was high (92.2%). However, the proportion of correct responses was low, indicating that the children had difficulties inhibiting saccades toward the distractor, and saccading in the opposite direction at target onset. The average proportion of correct saccades was 0.23 for 9-year-olds and 0.45 for 12-year-olds. The overall proportion of correct anti-saccades was 0.36, which is considerably lower than what would be expected in an adult population in a similar task. Success rates around 80% have been reported for adults in recent large-scale studies (Hutton and Ettinger, 2006). Conversely, saccade latencies in the child sample were longer than what would typically be expected among adults. In correct anti-saccade trials, the average saccade latency was 409 ms among 9-year-olds and 325 ms among 12-year-olds (overall 344 ms), while the same latency measure among adults typically lies around 200 ms (Holmqvist et al., 2011). Although we report saccade latency in the current study, only the individual proportion of correct anti-saccades was used as an independent variable in the statistical analyses. The reason for this is that the latter measure seems to have a higher validity with regard to children's gaze control.

### 3.2. Performance and distraction measures

**Figure 3:**
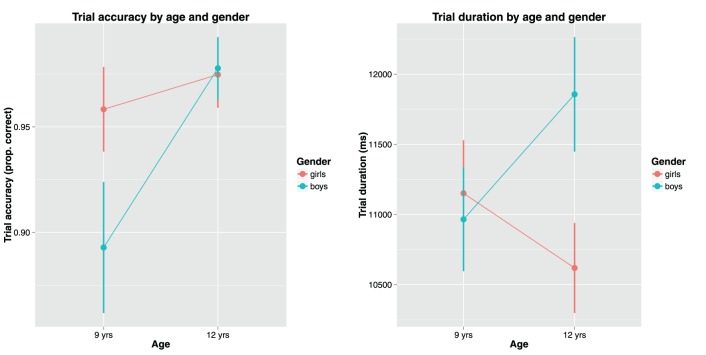
**Children's age and gender plotted against trial accuracy (left) and trial duration (right)**.

Task performance was measured as two product measures, trial accuracy (whether the task was answered correctly or not) and trial duration (the time taken to provide a solution to the tasks). All 36 trials contained valid performance data for all 45 participants. The overall trial accuracy was high (95.9% correct), but there was significant differences between 9-year-olds (93.4%) and 12-year-olds (97.6%), as well as between boys (95.1%) and girls (96.6%). Looking at trial duration, the average time to complete a task was just over 10 s (11558 ms). There was no significant difference in trial duration depending on age, but girls were about one second faster than boys on average. Trial number had a significant negative effect on trial duration, but no effect on trial accuracy, meaning that the children became faster to solve tasks toward the end of the experiment. Figure 3 shows the effect of children's age and gender on task performance.

**Figure 4:**
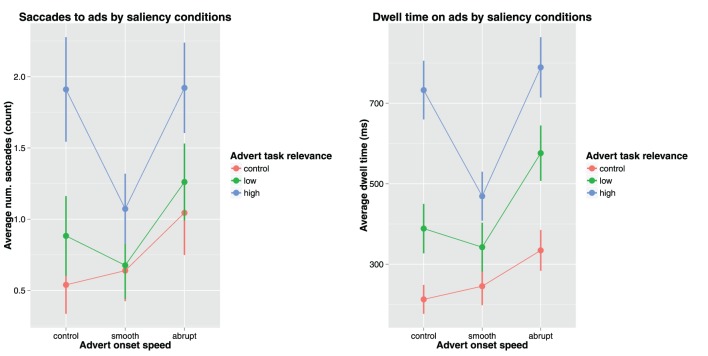
**Advert onset speed and task relevance plotted against saccades to ads (left) and dwell time on ads (right)**.

The distraction measures used in this study were dwell time on ads (attention retention) and saccades to ads (attention attraction). All 36 trials contained valid eye movement data for all 45 participants. The average fixation time on ads was just over half a second (654 ms), with no significant differences depending on age or gender. However, there was a significant negative effect of trial number, meaning that children tended to spend less time on experimental adverts toward the end of the experiment. The average number of saccades to ads was just over one saccade (1.13), and there was no significant differences depending on children's age or gender. As in the case of the previous distraction measure, there was a significantly negative effect of trial number, which would indicate that the children became less prone to behavioral distractions over the course of the experiment (as well as more proficient in solving the tasks). Figure 4 shows the effect of advert saliency conditions on task distraction.

### 3.3. Effects on task performance measures

We hypothesized that children's task performance would depend on individual factors as well as advert saliency conditions. More specifically, our hypotheses were that trial accuracy and trial duration could be described as a function of subject age and gaze control (H1a), level of advert onset speed (H1b), and level of advert task relevance (H1c). To test these hypotheses, the dataset was analyzed using linear mixed models in which the unique identifier of the experimental adverts was treated as a random factor (using the lme4 package in R). Subject was not entered as a random factor, since the gaze control variable also contained values that were unique for each participant. Fitting the data to these multi-level models provided partial support for our hypotheses regarding trial accuracy, but only weak support regarding trial duration. In the case of trial accuracy, all individual factors proved to have significant effects. Thus, older children as well as children with better gaze control were able to solve the tasks significantly more accurately, which gives support for hypothesis H1a. In the case of trial duration, the only significant effect was associated with male gender. Thus, boys generally required more time to solve the tasks. The advert saliency conditions did not seem to have a negative impact on trial accuracy or trial duration, and thus hypotheses H1b and H1c failed to gain support. Taken together, the evidence suggests that individual factors had an effect on one aspect of task performance (trial accuracy), while neither advert onset speed nor advert task relevance had any significant impact task performance.

Table 1 shows how advert saliency conditions and individual factors affected children's task performance. Task performance was divided into trial accuracy and trial duration, and the same independent variables were then used to model effects on both these performance measures. Tables for these performance measures are shown side by side. The coefficients and *p*-values for each independent variable are shown in the order they were entered. The advert saliency conditions consisted of three levels, and the effects of these conditions were tested aginst the control condition in the intercept. The level of multicollinearity between independent variables was low. In order to describe the model fit of the independent variables, the deviance of the proposed models were compared to the deviance of unconditional null models which included only the intercept and the random factor as independent variables. The proposed models and their corresponding null models were compared using chi-square tests, which showed that the independent variables contributed significantly to explaining the observed variance in trial accuracy and trial duration. Since the proposed models were used for hypothesis testing rather than modeling the best combination of predictors, no further attempts were made to optimize the models by excluding non-significant independent variables.

**Table 1:** **Effects of independent variables on task performance measures (trial accuracy and trial duration)**.

	**Trial accuracy (binomial)**				**Trial duration (ms)**			
	**Estimate**	**Std. error**	***z* value**	***Pr*(>|*z*|**)	**Estimate**	**Std. error**	***t* value**	***Pr*(>|*t*|**)
(Intercept)	2.8843	0.6755	4.2700	0.0000	10926.6540	520.9880	20.9730	0.0000
Advert onset speed (smooth)	0.3231	0.6324	0.5110	0.6094	8.3820	472.3650	0.0180	0.9858
Advert onset speed (abrupt)	−0.5470	0.5884	−0.9300	0.3525	435.4470	472.3400	0.9220	0.3567
Advert task relevance (low)	0.0166	0.5757	0.0290	0.9770	160.2250	472.2880	0.3390	0.7345
Advert task relevance (high)	0.8351	0.6290	1.3280	0.1843	−215.8810	472.2690	−0.4570	0.6477
Advert position (bottom-right)	**0.0108**	**0.0050**	**2.1380**	**0.0325**	−2.6040	3.2140	−0.8100	0.4179
Child age (12 years)	**0.9242**	**0.3275**	**2.8220**	**0.0048**	123.7080	215.0440	0.5750	0.5652
Child gender (male)	**−0.8124**	**0.2901**	**−2.8000**	**0.0051**	**653.6960**	**185.1890**	**3.5300**	**0.0004**
Child gaze control	**2.2361**	**0.7643**	**2.9260**	**0.0034**	−44.9860	471.4690	−0.0950	0.9240

Significant effects are emphasized with bold face. Child gaze control values have been centered.

### 3.4. Effects on task distraction measures

We also hypothesized that individual factors and advert saliency conditions would have distractive effects on children's gaze behavior while processing the tasks. The distraction measures that we analyzed in this study were: (1) dwell time on ads, and (2) saccades to ads, and we hypothesized that these measures would be sensitive to subject age and gaze control (H2a), level of advert onset speed (H2b), and level of advert task relevance (H2c). To test these hypotheses, additional linear mixed models were constructed using the lme4 package in R, in which adverts where treated as a random factor. As in the previous models, subject was not entered as a random factor, since the gaze control variable also contained values that were unique for each participant. Fitting the data to these multi-level models provided strong evidence for our hypotheses concerning both task distraction measures. Advert onset speed and advert task relevance were associated with increases in both dwell time on ads and saccades to ads. Thus, higher levels of advert saliency caused increased attentional retention as well as increased attention attraction in children, which provides support for H2b and H2c. Overall, there was a significant decrease on both distraction measures among children with better gaze control, which gives partial support for H2a. Contrary to H2a, the results for dwell time on ads show that older children spent significantly more time on experimental ads than younger children, but no such effect was detected in saccades to ads. Children's gender did not have any significant effects on distraction measures. Taken together, this evidence suggests that advert saliency conditions had a stronger effect on task distraction measures than individual factors, but better gaze control in children was associated with less distraction.

Table 2 shows how advert saliency conditions and individual factors affected children's task distraction. Task distraction was divided into dwell time on ads and saccades to ads, and the same independent variables were then used to model effects on both these distraction measures. Tables for these performance measures are shown side by side. The coefficients and *p*-values for each independent variable are shown in the order they were entered. The level of multicollinearity between independent variables was low. In order to describe the model fit of the independent variables, the deviance of the proposed models were compared to the deviance of unconditional null models in which all independent variables were excluded except the random factor. The proposed models and their corresponding null models were compared using chi-square tests, which showed that the independent variables contributed significantly to explaining the observed variance in both distraction measures. Since the proposed models were used for hypothesis testing rather than modeling the best combination of predictors, no further attempts were made to optimize the models by excluding non-significant independent variables.

**Table 2:** **Effects of independent variables on task distraction measures (dwell time on ads and saccades to ads)**.

	**Dwell time on ads (ms)**				**Saccades to ads (count)**			
	**Estimate**	**Std. error**	***t* value**	***Pr*(>|*z*|)**	**Estimate**	**Std. error**	***t* value**	***Pr*(>|*z*|)**
(Intercept)	485.9071	68.1109	7.1340	0.0000	0.8091	0.1543	6.7270	0.0000
Advert onset speed (smooth)	−78.8628	52.8385	−1.4930	0.1358	**−0.3115**	**0.1195**	**−2.3970**	**0.0166**
Advert onset speed (abrupt)	**125.8762**	**52.8353**	**2.3820**	**0.0173**	**0.2992**	**0.1194**	**3.0960**	**0.0020**
Advert task relevance (low)	**185.6223**	**52.4182**	**3.5410**	**0.0004**	**0.2047**	**0.1195**	**2.1060**	**0.0353**
Advert task relevance (high)	**412.5676**	**52.8994**	**7.7990**	**0.0000**	**0.8984**	**0.1194**	**8.4320**	**0.0000**
Advert position (bottom-right)	**−3.4347**	**0.5302**	**−6.4780**	**0.0000**	−0.0016	0.0012	−1.3800	0.1677
Child age (12 years)	**92.3068**	**35.5833**	**2.5940**	**0.0096**	−0.0267	0.0805	−0.1530	0.8786
Child gender (male)	−14.0102	30.5717	−0.4580	0.6468	0.1700	0.0692	1.6180	0.1058
Child gaze control	**−265.1405**	**77.8884**	**−3.4040**	**0.0007**	**−0.3102**	**0.1764**	**−2.4340**	**0.0151**

Significant effects are emphasized with bold face. Child gaze control values have been centered.

## 4. Discussion

We have tested the effects of advert saliency conditions on children's internet use while controlling for individual factors. The reported effects are a result of fitting observational data to the statistical model specified by our hypotheses. The main findings on children's task-oriented internet use are as follows: (1) Individual factors such as age, gender and level of gaze control have clear effects on both performance measures as well as distraction measures associated with solving the tasks; (2) Advert onset speed and advert task relevance only have a marginal effect on task performance, but have a clear effect on task distraction. A possible interpretation of these results is that children between 9 and 12 years of age are sensitive to advert saliency conditions on a behavioral level, but are still able to compensate for (or cope with) this distraction on a higher cognitive level, and consistently produce accurate responses during task-oriented internet use.

### 4.1. Individual factors and task-oriented internet use

When focusing on task-oriented internet use in relation to individual differences, a general pattern emerges revealing that individual factors tend to have a more profound impact on performance measures such as trial accuracy and trial duration (supporting H1a), than on distraction measures such as dwell time on ads and saccades to ads (disproving H2a). This difference is seen most clearly when looking at the gender variable, which shows that male gender affects both trial accuracy and trial duration negatively, whereas gender does not have any significant effects on distraction measures. In other words, boys had more difficulty solving the tasks than girls, and boys also needed more time to complete the tasks. However, in terms of distraction measures, boys and girls showed no differences. Looking at the age factor, the results give partial support for our hypotheses in that older children were associated with higher scores on trial accuracy (supporting H1a), but contrary to our expectations, older children were also associated with a significant increase in fixations on adverts (disproving H2a). Thus, older children unsurprisingly performed better than younger children on task accuracy, but children in the older age group also spent more time looking at the adverts. A possible interpretation of this pattern would be that older children have developed a better working memory, enabling them to engage in longer “detours” of attentional distraction, while still keeping track of the task at hand and produce accurate answers.

Still looking at individual factors, the strongest predictor of task performance and task distraction was not age or gender, but gaze control. In this study, gaze control was measured as children's ability to inhibit reflexive eye movements in an anti-saccade task. High scores on gaze control were clearly associated with higher scores on task accuracy (supporting H1a) and lower scores on both distraction measures (supporting H2a). The implication of these results is that children with better gaze control are more able to focus on the actual web-based task at hand while avoiding being distracted by salient internet adverts in the periphery. This interpretation fits nicely with other psychological research that has found strong positive correlations between gaze control and cognitive functions, e.g., working memory (Eenshuistra et al., 2004). According to the experimental design of the current study, we have chosen to investigate the age, gender and gaze control factors independently with regard to the dependent measures. The evidence suggests that children's individual level of gaze control plays an important role as a predictor of task performance and advert distraction. However, these results open up to other interesting lines of research in which the combined effects of these individual factors could be studied more carefully. Such a research direction could allow us to pinpoint various sub-groups among children that are particularly sensitive to advert saliency. For example, gaze control might develop differently in boys and girls, and by examining an interaction between age and gender with regard to gaze control, vulnerable sub-groups might be identified. Also, the contribution of motivational factors on task performance should be addressed in future research.

### 4.2. Advert saliency and task-oriented internet use

Contrary to our expectations, advert saliency conditions did not appear to affect performance measures in this study (disproving H1b and H1c). However, advert position, which was entered as a control variable, had a significantly positive effect on trial accuracy. This positive effect on task performance was associated with the bottom-right advert position, which was compared to the top-left position. The implication is that adverts that were placed in the top-left corner of the web page were associated with significantly more errors in trial accuracy, irrespective of advert saliency condition. This result applies directly to previous research on advertising effects where advert position has generated inconsistent results, mostly because of the fact that this property have been difficult to control for (Gidlöf et al., 2012). The present study can therefore conclude that adverts placed in the top-left corner of the web page are associated with a strong detrimental effect on task performance and a strong distractive effect in terms of total dwell time on adverts. A plausible explanation as to why the top-left advert position has a detrimental effect on web page interaction could be that this position tends to coincide with the typical starting position when reading text or when initiating a visual search task (Zelinsky, 1996).

In accordance with the expectations of this study, the advert saliency conditions proved to have strong effects on distraction measures (supporting H2b and H2c). Focusing first on advert onset speed and low-level visual features (H2b), our evidence suggests that abrupt onset speed in adverts caused a significant increase in dwell time on ads, as well as significantly more saccades to ads. That is, abrupt and dynamic visual features of internet adverts affect children's task-oriented internet use by causing distraction from the task, both in terms of attention retention and in terms of attention attraction. Slightly curiously, when investigating the effects of smooth advert onset speed the results tend to run counter to the hypothesized scenario. Thus, this dynamic visual feature is actually associated with less dwell time on ads and significantly less saccades to ads compared to the static control condition. Lower scores on these measures would mean that smooth advert onset speed causes reduced task distraction. To find these diametrically opposed behavioral effects caused by different levels of the same saliency factor (advert onset speed) is puzzling, and consequently this effect should be investigated further. One interpretation would be that smooth advert onset speed allows children to identify and avoid advert content through peripheral vision, while abrupt onset speed exerts a more coercive stimulus on the visual system, causing children to saccade toward the ads and also to fixate on the ads for an extended period of time. Recapitulating the arguments presented in the introduction, abrupt onset speed in adverts could represent a concrete example of “forced exposure” reported by previous internet advertising research done with children. Since advert onset speed is an objective and quantifiable visual feature, it seems to be an advertising property that could effectively be regulated in order to facilitate children's interaction with websites.

Changing focus to advert task relevance (H2c), the results show that this type of saliency causes more consistent and unequivocal effects on the distraction measures utilized in this study. Thus, both levels of advert task relevance were associated with significantly more dwell time on ads as well as significantly more saccades to ads. That is, pictorial advert content that is relevant to the task in some sense, affects children's task-oriented internet use by causing distractions from the task, both in terms of attention retention and in terms of attention attraction. Compared to the onset speed factor, advert task relevance thus appears to have a more powerful and detrimental effect on children's visual interaction with the web pages. These results are consistent with previous research on visual saliency, which have argued that task relevance have a more profound effect on gaze behavior and attention (Findlay and Walker, 1999). The downside of investigating task relevance is that these visual properties are considerably more difficult to define compared to low-level visual features. In the current study, low task relevance was operationalized as pictorial content that consisted of fake dialog windows, while high task relevance consisted of pictorial content that was similar to the target images that the children were solving the tasks for. The effects of these advert conditions were compared to pictorial content in a supposedly task irrelevant control condition. The problem with fake dialog windows and irrelevant advert content is that the subjective relevance of these types of pictorial content is difficult to control for since to some extent they depend on individual interests. Notwithstanding, the high task relevance condition had a more objective implementation in this experiment, and since this condition caused the strongest behavioral effects, we argue that we have successfully managed to document the effects of task relevance in internet adverts. Because of the difficulties in defining task relevance, it would probably be harder to regulate this aspect of internet adverts in order to facilitate children's internet use. The effects of task relevant advert content, could motivate further research into so-called behavioral targeting in advertising, in which web interaction metrics are collected in order to serve up more relevant adverts to users.

From a research perspective, it would be fruitful to develop the experimental paradigm used in the current study to include tests of other types of visual saliency in internet ads. We think that our study design offers a robust combination of ecological validity and experimental control that is well-suited for obtaining reliable and valid behavioral data on children's task-oriented internet use. Consequently, this design could easily be extended to investigate other eye movement measures such as fixation durations and blink rate. Developing our research in this direction would allow us to address questions about cognitive load in children when engaged in task-oriented internet use in commercial online environments. A limitation with the present study was the high overall scores on trial accuracy, which could indicate a ceiling effect on this measure. If the web-based tasks were too easy to solve accurately, then there would be less need for the children to compensate for attentional distractions in order achieve high task accuracy. In order to address the cognitive relationship between task distraction and task performance more thoroughly, we recommend increasing the task difficulty or limiting the trial duration.

From a policy and regulation perspective on online advertising, it is important to take these new findings into consideration when discussing possible restrictions of ads directed to children. Children in different age groups have repeatedly given verbal statements of how annoying, disturbing, and irritating online advertising is during their daily internet activities (Sandberg et al., 2011). Our study has provided empirical evidence that demonstrates how children's task-oriented internet use is disturbed by advertising saliency factors as well as advert positions. However, the children seem to cope with this distraction by adjusting their responses to accommodate for coercive advertising features and thus manage to compensate to some degree for these visual demands when involved in task-oriented internet use, i.e. children's task performance is adequate, but their experience of online advertising might be strenuous, especially for children that suffer from poor gaze control.

### Conflict of interest statement

The authors declare that the research was conducted in the absence of any commercial or financial relationships that could be construed as a potential conflict of interest.
