# Supplementary figures and images for: Advert saliency distracts children's visual attention during task-oriented internet use
Source: Front Psychol. 2014 Feb 12;5:51. doi: 10.3389/fpsyg.2014.00051 (PMC3921552; doi:10.3389/fpsyg.2014.00051)

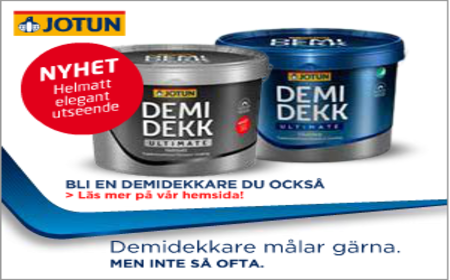

Supplement: Supplementary file 1 [file Presentation1.ZIP › con01.png]

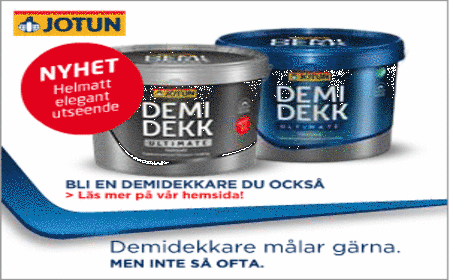

Supplement: Supplementary file 1 [file Presentation1.ZIP › con02.gif]

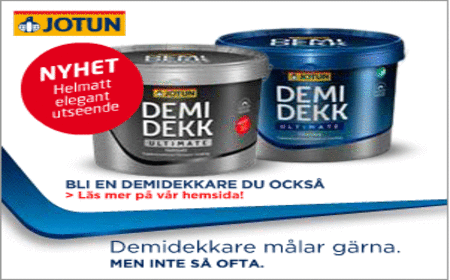

Supplement: Supplementary file 1 [file Presentation1.ZIP › con03.gif]
